# Traumatic Brain Injury: Novel Experimental Approaches and Treatment Possibilities

**DOI:** 10.3390/life15060884

**Published:** 2025-05-30

**Authors:** Kristina Pilipović, Tamara Janković, Jelena Rajič Bumber, Andrej Belančić, Jasenka Mršić-Pelčić

**Affiliations:** Department of Basic and Clinical Pharmacology and Toxicology, Faculty of Medicine, University of Rijeka, 51000 Rijeka, Croatia; tamara.jankovic@medri.uniri.hr (T.J.); jelena.rajic@medri.uniri.hr (J.R.B.); andrej.belancic@medri.uniri.hr (A.B.); jasenka.mrsic.pelcic@medri.uniri.hr (J.M.-P.)

**Keywords:** brain injuries, traumatic, brain–machine interfaces, nanomedicine, pluripotent stem cells, precision medicine, virtual rehabilitation

## Abstract

Traumatic brain injury (TBI) remains a critical global health issue with limited effective treatments. Traditional care of TBI patients focuses on stabilization and symptom management without regenerating damaged brain tissue. In this review, we analyze the current state of treatment of TBI, with focus on novel therapeutic approaches aimed at reducing secondary brain injury and promoting recovery. There are few innovative strategies that break away from the traditional, biological target-focused treatment approaches. Precision medicine includes personalized treatments based on biomarkers, genetics, advanced imaging, and artificial intelligence tools for prognosis and monitoring. Stem cell therapies are used to repair tissue, regulate immune responses, and support neural regeneration, with ongoing development in gene-enhanced approaches. Nanomedicine uses nanomaterials for targeted drug delivery, neuroprotection, and diagnostics by crossing the blood–brain barrier. Brain–machine interfaces enable brain-device communication to restore lost motor or neurological functions, while virtual rehabilitation and neuromodulation use virtual and augmented reality as well as brain stimulation techniques to improve rehabilitation outcomes. While these approaches show great potential, most are still in development and require more clinical testing to confirm safety and effectiveness. The future of TBI therapy looks promising, with innovative strategies likely to transform care.

## 1. Introduction

Traumatic brain injury (TBI) is one of the leading causes of death and disability worldwide, with significant social and economic implications. Each year, millions of individuals suffer from TBI, and many experience long-term impairments, including cognitive dysfunction, motor deficits, and emotional disturbances. Despite advancements in medical care, the management of TBI remains challenging, and effective treatment options are still limited. Current clinical approaches primarily focus on stabilizing the patient, preventing further injury, and alleviating symptoms, but there is no definitive therapy to repair or regenerate damaged brain tissue. This gap underscores the urgent need for innovative treatment strategies that can address the underlying pathophysiological processes of TBI and improve patient outcomes [1,2].

TBI is a complex condition that involves both primary and secondary injuries. The primary injury occurs at the moment of impact, leading to direct mechanical damage to brain tissue, blood vessels, and neuronal structures. However, it is the secondary injury processes that contribute significantly to the long-term consequences of TBI. These processes include neuroinflammation, oxidative stress, excitotoxicity, and disrupted blood–brain barrier integrity. In addition, a significant increase in the presence of “reactive astrocytes”, which serve as key regulators in both tissue damage and potential repair, was described. Understanding the intricate molecular interactions following a TBI, involving neurons, glial cells, and vascular networks, is crucial for managing the injury and its consequences. As a result, secondary injury can lead to progressive neuronal death, exacerbating functional deficits. The complexity of these mechanisms makes TBI difficult to treat, and current therapies, such as surgical interventions or pharmacological agents, are often only partially effective [3].

In recent years, there has been an increasing focus on novel experimental approaches aimed at mitigating secondary brain injury and promoting recovery. These strategies target various aspects of the injury process, from protecting neurons to stimulating tissue regeneration. Among the most promising approaches are neuroprotective agents that seek to limit cellular damage and preserve brain function. Additionally, stem cell therapies hold great promise, offering the potential to repair damaged tissue, regenerate neurons, and restore lost functions. Gene therapies, another innovative strategy, aim to modify cellular processes at the genetic level to promote healing and regeneration. Biomaterial-based interventions, such as scaffolds and hydrogels, are also being explored to provide structural support and guide tissue repair [4,5].

Emerging technologies are further expanding the therapeutic landscape for TBI. Nanotechnology, for instance, offers the ability to deliver targeted treatments at the cellular or molecular level, improving drug delivery and reducing side effects [6]. Brain–machine interfaces are being investigated for their potential to restore lost neurological functions through direct interaction with the brain. Additionally, the field of precision medicine holds great promise for tailoring treatment plans based on individual patient characteristics, enhancing the effectiveness of interventions [1,7,8]. 

Despite the exciting potential of these novel therapies, they are still in the experimental stage. The preclinical studies and experimental models, including animal models and in vitro systems, have provided valuable insights into the mechanisms underlying TBI and its recovery. However, clinical translation remains a significant hurdle, with many therapies yet to demonstrate their safety and efficacy in human trials. 

This review aims to explore the current experimental approaches to TBI treatment, focusing on novel therapies that have the potential to transform clinical practice. We will discuss the mechanisms of TBI, the strategies under investigation, and the challenges faced in developing effective treatments. By synthesizing the latest research, we aim to provide a comprehensive overview of the future possibilities for treating TBI and improving outcomes for patients worldwide.

## 2. The Role of Precision Medicine in the Traumatic Brain Injury Management

Despite significant advances in acute care, TBI remains a highly heterogeneous condition with unpredictable long-term outcomes. Traditional approaches to TBI management rely on generalized treatment guidelines that fail to account for individual variability in injury mechanisms, underlying pathophysiology, and patient-specific factors, such as genetics, comorbidities, and biomarker profiles. Precision medicine seeks to address this gap by leveraging an individualized approach that integrates molecular biomarkers, advanced neuroimaging, and machine learning (ML) techniques to tailor diagnosis, prognosis, and treatment to the unique characteristics of each patient [1,7].

One of the cornerstones of precision medicine in TBI management is the identification of reliable biomarkers that provide objective measures of injury severity, guide therapeutic decision-making, and predict long-term outcomes. Several biomarkers have emerged as potential candidates, including glial fibrillary acidic protein (GFAP), ubiquitin carboxyl-terminal hydrolase L1 (UCH-L1), neurofilament light chain (NfL), S100 calcium-binding protein B, and tau proteins [1,9]. GFAP and UCH-L1, for instance, have been shown to correlate with the presence of intracranial injuries on computed tomography (CT) scans and are currently included in U.S. Food and Drug Administration (FDA) approved diagnostic assays, such as the Banyan Brain Trauma Indicator [10,11,12]. Similarly, elevated levels of NfL have been associated with axonal injury and poor functional recovery, while tau proteins serve as potential indicators of neurodegeneration, particularly in repetitive head trauma cases [13,14]. The integration of these biomarkers into clinical practice offers a non-invasive means of risk stratification, enabling clinicians to tailor treatment strategies based on molecular profiles rather than relying solely on conventional imaging and clinical assessment tools.

Beyond biomarkers, genetic polymorphisms have been identified as key determinants of TBI susceptibility and recovery trajectories [7]. Among these, the apolipoprotein E (APOE) ε4 allele has been extensively studied for its role in modulating neuroinflammatory responses, blood–brain barrier integrity, and cognitive resilience following brain injury [15]. Carriers of the APOE ε4 allele have been shown to exhibit poorer recovery outcomes, increased risk of post-traumatic neurodegeneration, and higher susceptibility to chronic traumatic encephalopathy (CTE) compared to non-carriers [16,17]. Other genetic factors, including polymorphisms in brain-derived neurotrophic factor (BDNF) and tau genes, have also been implicated in differential recovery patterns following TBI [7]. These findings underscore the need for genotype-driven therapeutic strategies, such as targeted neuroprotective agents or individualized rehabilitation protocols, that align with the patient’s genetic predisposition.

Genome-wide association studies continue to refine genetic risk profiling by identifying loci linked to differential recovery trajectories, though challenges remain in standardizing phenotype definitions and controlling for the heterogeneity of injury mechanisms. Epigenomic alterations, including DNA methylation, histone modifications, and microRNA regulation, play a crucial role in modulating gene expression after TBI, influencing neuroplasticity, neuroinflammation, and secondary injury cascades. These dynamic, environment-sensitive modifications highlight the potential for epigenetic therapies but also introduce complexities in identifying stable biomarkers for clinical translation. Transcriptomic analyses provide further insight into injury-induced shifts in RNA expression, revealing dysregulated inflammatory and metabolic pathways that may serve as therapeutic targets. Notably, gene expression changes in peripheral leukocytes (OR11H1 and OR4M1) have shown promise as surrogate markers of brain injury severity and prognosis, offering a minimally invasive alternative to direct brain tissue analysis [18]. Standardizing data collection across multi-center studies and integrating multi-omics approaches will be critical for translating these findings into precision medicine strategies for TBI management [1].

Another key aspect of precision medicine in TBI management is the advancement of neuroimaging modalities that allow for a more refined assessment of brain injury. Conventional imaging techniques, such as CT and standard magnetic resonance imaging (MRI), have limitations in detecting subtle structural and functional changes, particularly in cases of mild TBI (mTBI) [1]. Emerging imaging techniques, including diffusion tensor imaging (DTI), susceptibility-weighted imaging, and functional MRI (fMRI), have demonstrated superior sensitivity in identifying microstructural abnormalities, axonal injury, and altered brain connectivity patterns that are often missed by traditional scans [19]. DTI, for example, provides insights into white matter integrity by measuring fractional anisotropy, which is crucial in detecting diffuse axonal injury—a hallmark of TBI that strongly correlates with long-term cognitive impairment. Similarly, fMRI has been used to assess functional connectivity disruptions in TBI patients, providing a potential tool for predicting neurocognitive outcomes and guiding rehabilitation strategies [19,20].

The integration of ML and artificial intelligence (AI) into TBI management has further enhanced the potential of precision medicine [20]. ML algorithms have been applied to neuroimaging data to automate lesion segmentation, quantify hematoma volumes, and predict long-term outcomes based on imaging-derived biomarkers [20,21]. AI-powered models have also been developed to analyze multimodal clinical data, including patient demographics, lab results, neurophysiological parameters, and imaging findings, to generate individualized prognostic predictions [22]. For instance, convolutional neural networks have shown promising results in detecting intracranial hemorrhages on CT scans with accuracy comparable to expert radiologists [23]. Moreover, predictive analytics using artificial intelligence (AI) has been employed to assess risk factors for secondary brain injury, enabling early intervention strategies to mitigate long-term complications.

Multimodal neuromonitoring represents another critical advancement in precision TBI management. Traditional approaches to intracranial pressure (ICP) monitoring have been supplemented by real-time assessments of cerebral autoregulation, brain tissue oxygenation (PbtO2), and metabolic changes using micro-dialysis [24]. These techniques provide a more comprehensive understanding of the pathophysiological mechanisms underlying secondary injury processes, allowing for individualized therapeutic adjustments. For example, personalized ICP thresholds based on pressure reactivity index monitoring have been proposed to optimize cerebral perfusion pressure management and improve outcomes in severe TBI patients [25]. Additionally, micro-dialysis-based monitoring of lactate/pyruvate ratios and glucose metabolism has been explored as a means of guiding neuroprotective strategies [26].

Despite these promising advancements, several challenges remain in translating precision medicine into routine clinical practice. One of the primary barriers is the standardization and validation of biomarker assays, neuroimaging protocols, and AI-based predictive models across diverse patient populations. Large-scale multicenter studies are needed to establish clinically relevant thresholds for biomarker interpretation and to refine ML algorithms for real-world applicability. Ethical considerations related to genetic testing, data privacy, and AI-driven decision-making also warrant careful attention to ensure equitable and responsible implementation.

## 3. Cell-Based Therapies for the Treatment of Traumatic Brain Injury: Neuroprotective and Regenerative Potential of Stem and Progenitor Cells

Cell transplantation therapies using various stem cell types have garnered significant attention as a promising treatment for TBI, offering potential benefits, such as neural repair, reduced inflammation, and improved functional recovery (Table 1). In addition to their therapeutic potential, stem cells can also be employed to investigate fundamental pathological processes, advancing our understanding of the mechanisms underlying TBI.

Stem cells are distinctive cells capable of self-renewal and differentiating into various specialized cell types, highlighting their multipotent nature. Initially, these cells can modulate the neural microenvironment, deposit extracellular matrix and release neurotrophic factors (BDNF, glial cell line-derived neurotrophic factor, insulin-like growth factor 1) that support the repair of damaged neurons [27]. Moreover, it has been shown that the stem cell-derived secretome, encompassing both diffusible molecules and extracellular vesicles, modulates the inflammatory response following TBI, thereby contributing to a more favorable immune environment that supports tissue repair [28]. Over time, they can promote nerve tissue regeneration by differentiating or transdifferentiating into mature neural cells [29,30]. Several cell types have been explored for post-TBI therapy, including neural stem cells (NSCs) from both adult and embryonic sources, induced pluripotent stem cells (iPSCs), and mesenchymal stem cells (MSCs) derived from adipose tissue, bone marrow, and umbilical cord [31]. 

**Table 1:** Selected recent preclinical studies on stem cells used to improve and investigate traumatic brain injury outcomes.

Type of Stem Cells	Main Results	Reference
Amnion-derived neural stem-like cells (AM-NSCs)	AM-NSC transplantation significantly improved neurological function and brain tissue morphology in a rat TBI model compared to AMSC and Matrigel controls. Effect was associated with enhanced expression of neurotrophic factors, despite low graft survival and minimal differentiation into neural-like cells.	[30]
Embryonic stem cell (ESC)-derived NSCs	Transplantation of human ESC-derived NSCs into immunodeficient TBI rats led to long-term cognitive improvement (≥2 months), particularly in hippocampal-dependent spatial memory, despite no change in lesion volume. Surviving NSCs (9–25%) differentiated into neurons, astrocytes, and oligodendrocytes, and cognitive recovery correlated with increased host hippocampal neuron survival.	[32]
Induced pluripotent stem cells (iPSC)-derived neurons	Mild stretch injury modeling mild TBI in human iPSC-derived neurons triggered amyloidogenic processing of APP, disrupting axonal transport and leading to accumulation of amyloid-related components associated with Alzheimer’s disease. Pharmacological inhibition of APP cleavage, as well as expression of the Alzheimer’s disease-protective A673T variant, prevented these stretch-induced transport defects, suggesting a potential strategy to reduce Alzheimer’s disease risk following mTBI.	[33]
Neural stem cells (NSCs)	Intracranial transplantation of clinical-grade fetal human NSCs in athymic rats with penetrating TBI showed no evidence of tumorigenicity or oncogenic tissue necrosis after six months, supporting the safety of human NSC therapy. Despite robust human NSC engraftment and predominantly neuronal differentiation of human NSCs into immature neurons, lesion size remained unchanged in athymic rats, highlighting a potential role of thymus-derived immune cells in modulating post-traumatic inflammation and tissue repair.	[34]
ESC-derived cerebral organoids	Transplantation of 8-week-old human embryonic stem cell-derived cerebral organoids (hCOs) in a mild TBI mouse model reduced neuronal death, enhanced neurogenesis and angiogenesis, and promoted repair of damaged cortical and hippocampal regions. hCO treatment improved cognitive function post-injury, supporting its therapeutic potential for neuronal dysfunction through cortical reconstruction and hippocampal neurogenesis.	[35]
iPSC-derived neural stem/progenitor cells (NS/PCs)	Genome-edited human iPSC-derived NS/PCs expressing yCD–UPRT enhanced motor recovery and reduced secondary brain injury, atrophy, and ventricle enlargement in a TBI mouse model. The yCD–UPRT/5-FC system enabled selective ablation of undifferentiated cells, preventing tumorigenesis while preserving surrounding neuronal tissue, improving the safety of iPSC-based therapy.	[36]
NSCs genetically modified to express human L-myc gene	Intranasally delivered L-myc-expressing human NSC (LMNSC008) migrated along white matter tracts to both primary and secondary injury sites in a rat TBI model. LMNSC008 treatment modulated gene expression by downregulating inflammatory pathways and microglial activation, supporting neuroprotection and tissue regeneration.	[37]

Abbreviations: ADSCs, adipose derived mesenchymal stem cells; AMSC, amnion derived mesenchymal stem cells; AM-NSC, amnion derived neural stem-like cells; APP, amyloid precursor protein; ESC, embryonic stem cell; iPCS, induced pluripotent stem cells; MSC, mesenchymal stem cells; mTBI, mild traumatic brain injury; NSC, neural stem cells; NS/PC, neural stem/progenitor cells; yCD–UPRT, yeast cytosine deaminase-uracil phosphoribosyl transferase.

MSCs play a crucial role in repairing damaged brain tissue through trophic support and immune regulation, activating pro-survival signaling pathways, inhibiting apoptosis, and enhancing neuroplasticity by promoting neurite outgrowth and synaptogenesis, ultimately leading to improved structural integrity and behavioral outcomes [38]. Recent studies indicate that MSCs derived from bone marrow effectively enhance motor recovery following TBI [39]. Additionally, they have been reported to attenuate injury-induced excitotoxicity by downregulating the membrane expression of glutamate receptors and modulating their downstream signaling pathways, a mechanism that may also play a critical role in mitigating secondary injury processes following TBI [40]. A major limitation of MSCs is their limited ability to differentiate into stable neural tissue. As a result, functional recovery relies largely on the secretion of neurotrophic factors during the acute phase of treatment [41]. 

In contrast, NSCs have the ability to differentiate into neurons and glial cells, promoting long-term functional recovery by restoring neural structures after brain injury [42,43]. Preclinical studies in TBI animal models have demonstrated that human NSCs can engraft, survive, and differentiate into neurons, capable of integrating into neural networks. In addition, they can secrete trophic factors, remyelinate damaged axons, and deposit extracellular matrix scaffolds, all of which contribute to improved neurological recovery and enhanced cognitive and motor functions [32,44,45]. Clinical trials have examined the potential of human NSCs derived from fetal cortical brain tissue or the spinal cord for ischemic stroke treatment [46]. However, their clinical application remains constrained by ethical considerations surrounding the use of human embryonic tissue, challenges associated with their procurement, and safety concerns related to the potential for tumorigenesis [31,34].

As a promising alternative, iPSCs potentially overcome ethical and logistical barriers [47]. They are generated in vitro from adult human cells, such as skin fibroblasts or bone marrow, through reprogramming with specific transcription factors or nuclear transfer [48]. To date, the therapeutic efficacy of these cells in experimental models of brain injury has been limited. iPSCs often retain epigenetic and transcriptomic signatures from their cell of origin, which may hinder their full differentiation into the desired cell type [49]. These limitations also raise concerns about tumorigenicity, posing significant safety challenges for iPSC-based regenerative medicine [50,51]. Gene-directed enzyme prodrug therapy offers a promising strategy to mitigate the risk of tumorigenesis [51]. In a study by Imai et al. [36], a novel gene therapy for TBI was developed using NS/PCs designed with a safeguard, i.e., a suicide gene, to eliminate undifferentiated cells after transplantation while preserving the adjacent neuronal structures. Furthermore, the study demonstrated that NS/PCs transplantation significantly improved motor function and prevented brain atrophy in a mouse model of TBI. 

Chaves et al. [33] utilized neurons derived from human iPSCs and used a custom-microfabricated device to replicate the mild stretch which neurons experience during mTBI. Their findings revealed that mechanical stress induces amyloid precursor protein (APP) cleavage, triggering amyloid β peptide generation and subsequently disrupting APP axonal transport. Notably, preventing APP cleavage preserved axonal transport integrity and prevented aberrant APP accumulation in axons. These findings provide valuable insights into the mechanisms linking mTBI to Alzheimer’s disease and may contribute to strategies for mitigating the associated risks.

Clinical translation remains elusive due to the complexities of iPSC generation, high production costs, and unresolved technical limitations. Moreover, ethical concerns persist regarding donor consent, the potential misuse of iPSCs in human cloning or germline modification, and the unknown long-term consequences associated with somatic cell reprogramming. Most clinical trials investigating stem cell therapies for TBI remain in early phases, with only a few reporting short-term safety and feasibility. Key challenges include limited long-term follow-up, lack of data on implantation efficiency and cell survival, and the need for more sensitive outcome measures to detect meaningful functional improvements [52].

## 4. Nanomedicine in Traumatic Brain Injury: The Use of Nanomaterials for Targeted Drug Delivery, Neuro-Regeneration, and Tissue Engineering

The complexity and heterogenicity of post-TBI pathological processes have posed significant challenges to developing effective disease-modifying therapies. In recent years, nanotechnology has been placed in the focus as an innovative neuroprotective TBI treatment [6,53]. There are various modalities of the use of nanomaterials after TBI (Table 2), as these materials can be used as carriers for small [54,55] and large molecules [56,57,58,59,60,61], chemicals [62,63,64,65], coding and non-coding nucleic acid molecules [66,67,68,69,70], gasses [71], drugs [72,73,74,75,76,77] and stem cells [70]. Furthermore, nanomaterials can be used as scaffolds to improve CNS regeneration [78,79], or as sealants to avoid tissue constriction [80]. Certain nanomaterials, i.e., exosomes, have experimentally showed intrinsic effect [66,81,82,83,84,85,86]. Nanomaterials can also be used in diagnostics as detectors of serum markers [87,88], or to facilitate imaging [86,89,90,91,92].

### 4.1. Use of Nanomaterials for Targeted Drug Delivery 

Nanomaterial morphology plays a critical role in pharmacokinetic behavior and blood–brain barrier penetration and it is identified as a key parameter in the design of CNS-targeted drug delivery systems [93]. Comparing to ordinary drugs, nanomaterial absorption varies significantly [6] and highly depends on the administration route, as intravenous delivery allows direct entry into systemic circulation, while subcutaneous or intramuscular routes involve lymphatic transit before bloodstream entry. A critical requirement for a nanomedicine targeting brain after TBI is the ability to cross the blood–brain barrier (BBB), which is generally more permeable following injuries of varying severity [94]. For instance, numerous proteins have demonstrated a significant therapeutic potential for the treatment of complex neurological disorders such as TBI. A study by Waggoner et al. [69], showed that BDNF loaded onto biodegradable porous silicon nanoparticles enhances BBB penetration and significantly reduces lesion volume. Tailoring nanomaterial properties offers a promising strategy to overcome the pharmacokinetic limitations of traditional drugs and offers improvement in targeted drug delivery to injured brain regions. Before nanomaterial application, studies on size-dependent biodistribution, BBB penetration stability and microenvironment responsiveness should be examined. Nevertheless, critical challenges remain in optimizing biocompatibility, systemic clearance, and long-term safety profiles [6].

### 4.2. Nanoparticles: Polymeric and Metallic Nanoparticles and Nanogels

Nanoparticles (NPs) are drug delivery nanomaterials with high specificity to the target organ and rapid diffusion properties, with sizes less than 500 nm [6,95]. Polymeric NPs primary interact with immune and vascular cells, modifying the brain’s environment, and are often used as drug carriers in TBI [6], but also after spinal cord injury [96]. Most studies on polymeric NPs are focused on acute consequences, with promising results in neuroprotection and behavioral outcomes. Their potential impact in chronic TBI remains uncertain [97]. Attachment of polymers or functional groups on NPs’ surface, known as functionalization [96] (Table 3), greatly improves polymeric NP functions. Frequently used degradable polyesters are preferred due to their broad availability and prior approval [97], while conjugation of poly (ethylene) glycol is often applied in order to prolong NPs’ systemic circulation time by minimizing the absorption by the reticuloendothelial system [68,69]. Recent studies emphasize the potential of functionalized NPs for optimizing the delivery of therapeutic agents, including drugs, MSCs, and small interfering RNAs (siRNAs) [57,68,70,75]. These advancements enhance neural function, mitigate oxidative stress, exert anti-inflammatory effects, and ultimately improve neurological outcomes [55,60,79,98]. 

Nanogels are three-dimensional hydrogel structures formed by a crosslinked polymer mesh that can absorb water while staying insoluble. By addition of different active components, nanogel properties—e.g., size, shape, charge, porosity, flexibility and biodegradability—can be altered [99]. Nanogels can serve as a drug carriers for anti-inflammatory drugs [74,76], growth factors [61] and stem cells [70], and improve volume lesion site. The use of hydrophobic methacrylate gelatin nanogel as a sealant for structural support has shown a great potential in nervous system recovery [80].

Metallic NPs have attracted a significant interest due to their potential in visualization techniques, i.e., tracking stem cells [92]. They have demonstrated the ability to navigate through tissues more effectively than polymeric nanoparticles [97]. For example, cerium oxide metallic NPs, used as drug carriers, show antioxidant properties and improved recovery in experimental animals [63]. Furthermore, gold NPs can be used for detection of ultralow concentrations of serum biomarkers [87,88].

### 4.3. Lipid Nanoparticles

Lipid nanomaterials are considered highly promising for biological application due to their non-toxic lipid components, and have gained attention as an effective nanoscale drug delivery system, particularly in mRNA-based COVID-19 vaccines [100]. Recent studies highlight the use of lipid NPs for efficient delivery of drugs [73], MSCs, and siRNAs [69], enhancing neural function and reducing oxidative stress. Additionally, fluorescently labeled lipid nano-droplets have been detected in the penumbra of TBI mice, where they were engulfed by neurons [90].

Liposomes, currently the most commonly used nanocarriers due to their excellent biocompatibility, biodegradability, and low immunogenicity [101], are spherical nanocarriers built from hydrophobic lipid bilayer [6] and, if charged (i.e., with cations), express higher stability and reduced aggregation capability. They are used in gene therapy, as they are capable of transporting negatively charged biomolecules, such as DNA, RNA and oligonucleotides [102]. 

Leukosomes are proteolipid vesicles that contain phospholipids and membrane proteins derived from leukocytes and are used for drug delivery in the treatment of various disorders with few available therapeutic options, such as TBI. Functionally, leukosomes preserve the natural tendency of leukocytes to selectively bind to inflamed blood vessels in vivo, promoting tissue repair by maintaining structural integrity and limiting neutrophil infiltration [103]. Biomimetic NPs, such as liposomes and leukosomes, can be designed to carry a contrast agent that can penetrate into the inflamed brain area after TBI, and be used as a diagnostic tool [86]. Additionally, empty liposomes and leukosomes show a therapeutic effect by reducing lesion size, with higher adherence affinity to the lesion blood vessels in leukosomes than liposomes [86]. 

### 4.4. Exosomes

Exosomes are membrane bound vesicles that can encapsulate lipids, proteins, nucleic acid, messenger RNAs, non-coding RNAs and cytokines needed for normal cell communication. Exosomes have a great potential for therapeutic purpose [104], specifically in miRNA transport [105]. For example, exosomes from human platelet concentrates showed inflammation reduction capacity by lowering GFAP and tumor necrosis factor alfa mRNA levels [81], while umbilical cord MSC exosomes have enhanced neurological recovery through inhibition of the NF-κB pathway [85]. Studies have shown that endogenous neurogenesis can be stimulated with neuronal stem cell-derived exosomes preconditioned with interferon gamma [56], while umbilical cord MSC exosomes can stimulate neurogenesis, inhibit apoptosis and reduce inflammation [66]. Additionally, motor and cognitive function have been improved after intranasal delivery of adipose-derived stem cell exosomes [83] and microglia derived exosomes with miR-124-3p that target damaged hippocampal neurons [82].

### 4.5. Carbon Dots and Carbon Quantum Dots

Carbon dots and carbon quantum dots are semiconducting NPs [106] used for diagnostics, bioimaging and drug delivery purposes [107,108]. For example, intravenous administration of PEG-capped silver indium selenide-based quantum dots (QDs) has shown precise, real-time hemorrhage detection and surgical guidance in mice [89]. Improvement in imaging-guided treatment, with concomitant antioxidant activity has also been shown in the use of fluorescently labeled QDs with uniformly distributed manganese atoms [91]. Additionally, application of carbon dots functionalized with herbal medicines enables improvement in neurological functions, by reducing brain edema, neuronal damage, and BBB permeability after TBI [64]. Collectively, these nanomaterials combine diagnostic imaging and therapeutic antioxidant functions, offering promising integrated strategies for TBI management.

## 5. Innovations in Traumatic Brain Injury Rehabilitation: Brain–Machine Interfaces and Virtual Rehabilitation Interventions

Neurorehabilitation strategies for post-TBI recovery have traditionally been focused on the biological manipulation of cellular processes and disturbances, with the primary aim of promoting neuronal repair and neuroregeneration. However, achieving long-term functional recovery requires the use of alternative and innovative approaches, such as the construction of brain–machine interfaces (BMIs), also known as brain–computer interfaces as well as virtual rehabilitation interventions (VRIs) [109,110]. These methods target neuroplasticity in an effort to restore motor, cognitive, and psychosocial functions of affected individuals. Emerging techniques in neurorehabilitation include robotic-assisted therapy and exoskeletons, virtual reality (VR) and augmented reality (AR) applications, wearable technologies, and sensor-based systems [111]. In addition to these, neuromodulation is one of the emerging fields in neurology and psychiatry, but also in neurorehabilitation of patients with acquired brain injury [112].

### 5.1. Brain–Machine Interface-Driven Approaches in Traumatic Brain Injury Rehabilitation

BMIs are defined as systems that decode neural signals to enable direct communication between the brain and an external device [113]. These signals can be obtained through non-invasive or invasive methods and are then used to control external devices [114]. Neural or brain–machine interfaces use these signals, connected to computers that extract, interpret, and process the information from the nervous system to generate functional outputs. In the context of TBI rehabilitation, the functional outputs aim to promote motor recovery, such as controlling robotic exoskeletons [115,116,117], or cognitive retraining through neurofeedback [118]. 

**Figure 1:**
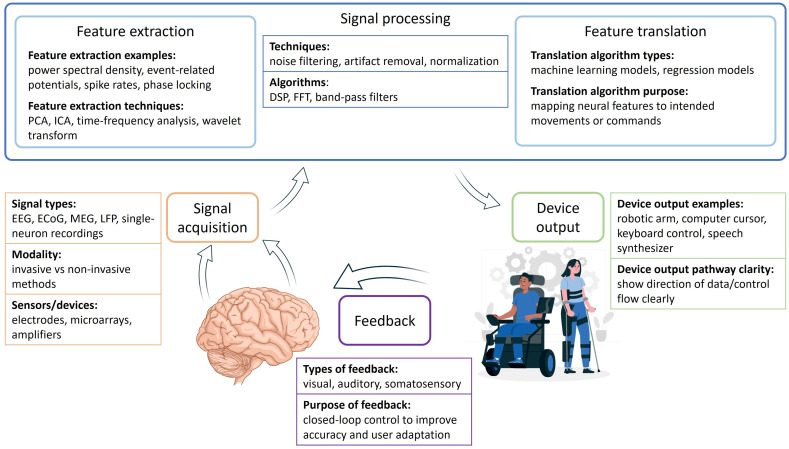
Diagram depicting the workflow of a brain–machine interface, comprising sequential stages: signal acquisition and processing, feature extraction and translation, device output, and feedback. Abbreviations: PCA, principal component analysis; ICA, independent component analysis; DSP, digital signal processing; FFT, Fast Fourier Transform; EEG, electroencephalogram; ECoG, electrocorticogram; MEG, magnetoencephalography; LFP, local field potential.

Main components of BMIs are signal acquisition, signal processing, feature extraction and translation, and the device output component (Figure 1). The first step in the process is the signal acquisition, i.e., collection of raw brain signals via sensors, which can involve non-invasive methods as well as invasive neural implants [114]. Non-invasive acquisition can be achieved by using electroencephalogram, magnetoencephalogram, or MRI to capture brain activity without penetrating the skull [119]. Invasive techniques include implantation of the intracortical electrodes that can record single-unit activity directly from neurons within brain tissue for the acquisition of high-resolution data [119]. In between these are the partially invasive techniques such as electrocorticography that measures signals directly from the cortical surface [120]. The next step is the preprocessing of the acquired raw brain signals, removal of noise and artifacts caused by environmental factors or physiological interference, and preparation of the obtained information for the feature extraction (i.e., analyses of the preprocessed signals to identify patterns or specific signal characteristics) and translation (i.e., translation of the extracted feature into commands using machine learning algorithms or other computational models) [121]. The final component, device output, is devised to execute the translated commands through an external device (e.g., prosthetic limbs, wheelchairs, robotic systems or machines which enable communication via speech synthesis or text input) [122]. Many BMI systems operate in a closed-loop format, i.e., the actions of the user generate feedback used for the necessary system adjustments [123,124]. 

In TBI rehabilitation, BMIs hold significant potential, enabling direct communication between the brain and external devices. The research summarized in Table 4 demonstrates the effectiveness of various BMI approaches in TBI patients, offering promising avenues for improving recovery and restoring lost functions. As research continues, BMIs could play a crucial role in advancing personalized rehabilitation strategies for TBI.

### 5.2. Virtual Rehabilitation Interventions in Neurorehabilitation

In neurorehabilitation, VRIs provide an immersive digital environment that uses head-mounted displays, motion sensors, or haptic feedback to simulate real-world tasks [125]. Key applications of VRIs in TBI patients are in motor skill reacquisition, anxiety reduction via exposure therapy, and memory enhancement through adaptive scenarios [125,126,127]. BMIs and VRIs can also be used as hybrid systems through which synchronization of BMI-derived neural data with VRI visual cues to accelerate skill relearning are combined [128]. Improvement of motor function, the development of cognitive abilities, and the stimulation of the senses are the main outcomes aimed in the use of VRI-based technologies [129]. VRIs often encompass VR and AR technologies [130]. Although VR and AR differ in their approach and level of immersion, they have been increasingly introduced as complementary therapeutic approaches in neurorehabilitation, including the treatment of TBI patients [110,131,132,133,134,135,136,137,138,139,140]. Beyond VR and AR, there are other new and emerging technologies and methodologies, which have been investigated to see if they can enhance rehabilitation outcomes (Table 4).

**Table 4:** Overview of immersive and assistive technologies in healthcare and summary of some recent clinical studies on their role in brain injury rehabilitation.

Technology Type	Definition and Key Features	Clinical Applications in Healthcare	Application in Brain Injury Rehabilitation
Virtual Reality (VR)	Creates a fully immersive digital environment that replaces the real world. Users interact with this environment through headsets, controllers, and other devices that track body movements and provide sensory feedback.	Widely used for motor skill retraining, cognitive therapy, and psychological interventions.	[132,134,135,141,142,143,144,145,146,147,148,149,150,151,152,153]
Augmented Reality (AR)	Overlays digital elements—such as images, videos, or 3D models—onto the real-world environment. Enhances reality without fully replacing it and is typically accessed through smartphones, tablets, or AR glasses.	Particularly useful for providing real-time guidance during physical therapy exercises or offering visual cues to improve motor coordination. It has also been used in cognitive training by overlaying interactive tasks onto physical spaces.	[130,137,138,145,154]
Video Capture VR	Uses cameras and software to track user movements without requiring physical markers on the body. The user’s image is embedded into a virtual environment, enabling natural interaction with animated graphics.	Useful for balance training, motor skill recovery, and functional movement exercises.	[155]
Interactive Video Gaming	Employs commercial gaming systems or custom-designed games to engage patients in therapeutic activities.	Often used for home-based rehabilitation, providing accessible and engaging platforms for motor skill training, cognitive exercises, and physical activity.	[156,157,158,159]
Tele-Rehabilitation	Uses high-speed networking to connect patients with therapists remotely. This enables virtual therapy sessions and real-time monitoring of rehabilitation progress.	Facilitates access to therapy for patients in remote areas or those with limited mobility. It can include virtual environments combined with haptic devices, video conferencing, and data analytics tools.	[136,160,161]
Behavior Change Techniques	Integrated into VRIs to promote behavior modification through structured interventions, such as goal setting, adaptability, feedback mechanisms, and competition.	Used within VR environments to enhance motor recovery by tailoring tasks to individual needs and providing explicit feedback on performance.	[162]
Wearable Sensors	Wearable devices equipped with sensors measure physiological responses such as movement patterns, muscle activity, or heart rate during rehabilitation exercises.	Provide real-time feedback and data collection for both therapists and patients, enabling personalized adjustments to therapy protocols.	[163,164,165]
Extended Reality (XR)	Encompasses VR, AR, and mixed reality, offering hybrid environments that combine digital elements with the physical world.	Increasingly used in rehabilitation to provide immersive yet contextually relevant environments for motor skill training, cognitive exercises, and social interaction.	[138,139,166]
Haptic Feedback Devices	Simulate tactile sensations by applying force or vibrations to the user’s skin or muscles.	Enhance the realism of virtual environments by providing physical feedback during tasks such as grasping objects or navigating virtual spaces.	[167,168]
Metaverse-Based Rehabilitation	Integrates hardware (e.g., XR devices) and software platforms (e.g., rendering tools) to create interconnected virtual spaces for rehabilitation.	Collaborative therapy sessions, gamified exercises, and continuous follow-ups post-discharge from clinical care.	[140]

### 5.3. Application of Neuromodulation Techniques in Brain Injury Recovery

One of the emerging fields in brain injury rehabilitation (but also medicine in general) is neuromodulation [112]. Neuromodulation technologies use stimulants, such as electricity, magnetism, sound, and light to modify the activity of neurons, either by exciting, inhibiting, or synchronizing neural activity, with the goal of enhancing or restoring function in the nervous system, but without causing damage [169]. Neuromodulation can be achieved by different approaches, including non-invasive, invasive, and a combined technique (Table 5). Even though many are still being researched, some have already been adopted into standard care treatments for various neurological and psychiatric conditions. For example, deep brain stimulation has been FDA-approved since the late 1990s [170], transcranial magnetic stimulation is used in major depressive and obsessive–compulsive disorders [171,172], vagus nerve stimulation helps patients with drug-resistant epilepsy since 1997 [173,174], and spinal cord stimulation has been shown to be effective for failed back surgery syndrome [175], complex regional pain syndrome [176], and diabetic neuropathy [177]. A comprehensive review of neuromodulation techniques explored in TBI rehabilitation has recently been done by Calderone et al. [112] and Kundu et al. [178]. Though there are some promising results from these studies, unlike in other neurological conditions (e.g., Parkinson’s and depression), in TBI patients there are specific safety issues [112]. Namely, as TBI itself can increase the risk of seizures, electrical stimulation can increase this risk. There is also higher risk of infection from implanted devices. Nevertheless, there are some new and emerging stimulation technologies that offer better control and specificity in neuromodulation therapy and that might make neuromodulation techniques a valuable tool in TBI neurorehabilitation [179].

### 5.4. Ethical and Regulatory Challenges Facing Brain–Machine Interface and Virtual Rehabilitation Interventions in Brain Trauma Care

With the emergence of new technologies, which offer promising advancements in brain trauma care, enabling neural recovery and enhanced functional outcomes, their integration raises significant ethical and regulatory challenges [199,200,201]. One major concern is informed consent, particularly with cognitively impaired patients, making it difficult to ensure voluntary and fully understood participation [202]. BMI systems raise unprecedented concerns about cognitive liberty due to worries of unauthorized use of neural data that could enable manipulation or discrimination. Additionally, there is the risk of technological dependency, where patients may become reliant on devices without long-term support or access, as well as reservations due to the high costs of these treatments that might cause social disparities and limit access to only certain groups of patients [203]. From a regulatory perspective, standardization and oversight are usually behind technological innovation that is also the issue with BMI, VR and similar technologies. Lastly, the long-term effects of brain-device interaction is still not adequately researched, with unclear long-term cognitive benefits for TBI. As these technologies advance, ethical frameworks and regulatory bodies must evolve in parallel to ensure responsible development, just access, and patient-centered implementation in brain trauma rehabilitation.

## 6. Conclusions and Future Considerations

In conclusion, the future of TBI therapy looks promising, with a range of innovative approaches being explored. Pharmacological and neuroprotective strategies, including multifunctional drugs, offer new avenues for preventing or mitigating damage. Precision medicine, powered by pharmacogenomics and “omics” technologies, enables more personalized and effective treatments. The advancement of cell-based therapies, particularly using human iPSCs, holds great potential for repairing brain damage at a cellular level. Lastly, emerging rehabilitation techniques such as BMIs and VRIs are revolutionizing recovery methods, offering patients new hope for rehabilitation and quality of life. These diverse therapies collectively represent a transformative shift in how TBI is treated and managed in the future.

Another opportunity to consider is the integration of molecular and cellular based novel treatment models with the recent innovative technological approaches. For example, combining molecular therapies (e.g., stem cells) with technological delivery systems (e.g., biomaterials) enhances tissue repair and functional recovery [204]. Nanoparticles and nanocarriers address the blood–brain barrier challenge, improving precision for gene therapy and anti-inflammatory agents [1], and techniques like photo-biomodulation and VR rehabilitation address chronic inflammation and cognitive deficits, complementing molecular interventions [204,205]. Structured comparison of innovative approaches in brain trauma treatment that integrate molecular and technology-based strategies targeting both primary injuries and secondary cascades is presented in Table 6 in which their mechanisms and synergies have been highlighted.

At present, the readiness of these innovative approaches for clinical implementation varies (Table 7). Nanomaterials have shown promise in preclinical studies, particularly in crossing the BBB and reducing secondary injury cascades; however, clinical validation is still lacking [206]. Cell-based therapies, particularly those using bone marrow-derived MSCs, have demonstrated regenerative and anti-inflammatory effects in early-phase trials, with therapeutic outcomes influenced by patient age [207]. Currently, biomarker-driven strategies remain in early development, primarily focusing on genetic subtyping to enable more personalized interventions [1], while BMI systems are progressing in clinical applications for motor impairment, whereas VR is still largely exploratory, particularly in the context of TBI-specific cognitive retraining [208].

Lastly, this review was intentionally designed as a narrative synthesis to provide a broad yet focused overview of emerging experimental approaches in TBI, rather than a systematic evaluation of individual clinical trials. Future systematic reviews, each focused on specific subtopics covered in this narrative review, will be essential to critically compare trial designs, methodologies, dosing strategies, adverse events, and clinical outcomes in a more granular and evidence-weighted manner.

## Figures and Tables

**Table 2:** Nanomaterials in traumatic brain injury models and their functions.

Function/Use	Type	References
Carriers	Small molecules	[54,55]
	Large molecules	[56,57,58,59,60,61]
	Chemicals	[62,63,64,65]
	Coding and non-coding	[66,67,68,69,70]
	Nucleic acid molecules	[71]
	Gasses data	[72,73,74,75,76,77]
	Drugs	[70]
	Stem cells	[78,79]
Scaffold		[80]
Sealant		[66,81,82,83,84,85,86]
Intrinsic effect		[87,88]
Diagnostics	Serum markers’ detection	[86,89,90,91,92]
	Imaging	[54,55]

**Table 3:** Selected recent studies on nanomaterials used to improve traumatic brain injury outcomes.

Nanomaterial	Surface Functionalization	Main Results	Reference
Polymeric nanoparticles (NP)	Poly (butyl cyanoacrylate) (PBCA)	PBCA NPs effectively deliver large molecules, such as HRP or EGFP, across the BBB 4 h post-TBI.	[57]
		PBCA NPs deliver β-NGF factor and promote the neurite outgrowth and reduce mortality after TBI in rats.	[54]
	Poly (lactic-co-glycolic acid) (PLGA)	Transplantation of PLGA scaffolds combined with MSCs/MSC and NGF enhances neural function and restores brain tissue structure in after TBI.	[79]
		PLGA with SOD1 and CAT reduce ROS post-TBI.	[60]
		PLGA based DOPA-NGF microunits improve neuronal recovery and decrease neuronal loss, astrocytic and microglial activation.	[55]
	Poly (ethylene) glycol (PEG)	PEG hydrophilic carbon cluster ameliorates neuronal loss, oxidative stress and repairs BBB.	[84]
		PEG prolongs RNA NPs’ half-life in blood in order to knockdown TNF-α cytokine, increase nanoparticle stability, reduce of protein adsorption and uptake by the reticuloendothelial system.	[67]
		Xenon-containing microbubbles functionalized with PEG reduce BBB disruption.	[71]
		PEG-azide modifies clotting cascade in in vivo response and increases neuronal survival in TBI rats.	[62]
	PLGA–PEG	PLGA-PEG NPs encapsulating isoliquiritigenin can replicate the effects of intracranial isoliquiritigenin administration in lowering serum COX-2 levels.	[75]
	PLGA–polysorbate (PLGA–PS)	Tau siRNA–loaded PS-NPs silence tau expression in vivo early and late after TBI.	[68]
Metallic NP		Cerium oxide NPs, coated with 6-aminocaproic acid and polyvinylpyrrolidone, show antioxidant properties and improve recovery post-TBI.	[63]
		Anti-S100 B labeled gold NPs can be used to determine concentration of S100B in human serum.	[87]
		Gold NPs in an optical microfiber interface increase sensitivity of ultralow concentrations of GFAP in human serum post-TBI.	[88]
		Superparamagnetic iron oxide NPs can serve as an MRI contrast agent for labeling MSCs, enabling non-invasive, real-time in vivo tracking following intranasal delivery post-TBi in mice.	[92]
Nanogel		PEG hydrogel containing dexamethasone-conjugated hyaluronic acid improves motor function, and reduces lesion volume and inflammation after mild and moderate TBI.	[74,76]
		Hyaluronic acid-based hydrogels based and gelatin combined with salvianolic acid B and VEGF, used as sealants, reduce the lesion volume site.	[61]
		Hyaluronic acid hydrogels encapsulating bone MSCs and nerve growth factor may enhance neurotrophic support and mitigate neuroinflammation, thereby facilitating neurological recovery and functional restoration post-TBI in mice.	[70]
		Hydrophobic methyl-acrylated gelatin mitigates TBI-induced mortality, neurological deficits, and cerebral edema while modulating iron-related toxicity via PI3K/PKC-α signaling.	[80]
Lipid NPs (LNPs)	PEG	LNPs are a promising non-viral gene therapy platform for treating TBI and PEG-LNPs prolong blood half-time after i.v. administration.	[69]
		Fluorescently dyed lipid nano droplets can be used to track nanocarriers in brains of TBI mice and are able to cross the BBB by endothelial transcytosis into the penumbra and are later internalized by neurons.	[90]
		A lipoprotein nanocarrier can deliver cyclosporine A to damaged brain sites, effectively reducing neuronal damage, alleviating neuroinflammation, and rescuing memory deficits post-TBI.	[73]
Liposomes		Baicalein encapsulated in liposomes decreases brain edema, reduces inflammatory cytokine serum levels and improves motor function outcomes.	[65]
		Dexamethasone encapsulated in liposomes can specifically affect the damaged brain, decreasing lesion size, neuronal loss, astrogliosis, pro-inflammatory cytokine release, and microglial activation predominantly in male mice acutely post-TBI.	[72]
		Liposome encapsulated with 20-hydroxyeicosatetraenoic acid inhibitor, applied i.v., reduces lesion volume, neuronal degeneration, microglial activation and ameliorates neurological outcome.	[77]
		Delayed application of intranasal liposomes with anti-inflammatory protein IL-4 preserves the structural and functional integrity of white matter via oligodendro-genesis, and facilitates long-term sensorimotor recovery.	[59]
		Liposomes applied i.v. can be used as imaging agents due to their ability to carry contrast agent and target inflamed brain area. Empty liposomes also reduce lesion volume and show therapeutic effect after experimental TBI in mice.	[86]
		VCAM-1 liposome nanocarrier concentrate in the brain at higher levels than untargeted IgG controls after intravenous injection.	[58]
Leukosomes		Leukosomes, applied i.v., serve as effective imaging agents by delivering contrast agents, specifically targeting inflamed brain regions. Additionally, empty liposomes have higher adherence affinity to lesion blood vessels and reduce lesion size following experimental TBI in mice.	[86]
Exosomes		Exosomes from human platelet concentrates’ supernatants show a strong anti-inflammatory effect by decreasing GFAP and TNFα mRNA levels after TBI in mice.	[81]
		Exosomes from umbilical cord MSCs enhance neurological recovery of TBI rats by NF-κB pathway inhibition.	[85]
		Exosomes derived from neural stem cells preconditioned with IFN-γ supports the regeneration of damaged neural tissue and enhances endogenous neurogenesis.	[56]
		I.v. application of exosomes from human umbilical cord MSCs can improve neurological repair after TBI in rats by inhibiting apoptosis, promoting neurogenesis and reducing inflammation.	[66]
		Intranasal administration of exosomes from human adipose-derived stem cells 48 h after injury alleviates motor and cognitive deficits following TBI in rats.	[83]
		I.v. injected microglial exosomes are absorbed by injured brain neurons, delivering miR-124-3p to hippocampal neurons, mitigating neurodegeneration and ameliorating cognitive recovery after rmTBI in mice.	[82]
Carbon dots and carbon quantum dots		I.v. application of PEG-capped silver indium selenide-based quantum dots enables precise hemorrhage diagnostics after TBI in mice.	[89]
		I.v. applied carbon dots, functionalized with herbal medicine, ameliorate neurological functions and reduce brain edema, neuronal damage and BBB permeability.	[64]
		Fluorescently labeled quantum dots enable imaging-guided treatment of TBI while exhibiting antioxidant activity due to uniform Mn atom distribution.	[91]

Abbreviations: NP, nanoparticles; PBCA, poly (butyl cyanoacrylate); HRP, horseradish peroxidase; EGFP, enhanced green fluorescent protein; BBB, blood–brain barrier, TBI, traumatic brain injury; β-NGF, β-nerve growth factor; PLGA, poly (lactic-co-glycolic acid); MSC, mesenchymal stem cells; NGF, nerve growth factor; SOD1, superoxide dismutase; CAT, catalase; ROS, reactive oxygen species; DOPA-NGF, 3,4-dihydroxyphenylalanine nerve growth factor; PEG, poly (ethylene) glycol; TNF-α, tumor necrosis factor alfa; COX-2, cyclooxygenase-2; PLGA-PS, PLGA-polysorbate; VEGF, vascular endothelial growth factor; PI3K/PKC-α, phosphatidylinositol 3-kinase/protein kinase C alpha; S100B, S100 calcium-binding protein B; GFAP, glial fibrillary acidic protein; MRI, magnetic resonance imaging; LNPs, lipid nanoparticles; IL-4, interleukin 4; VCAM-1, vascular cell adhesion protein 1; IFN-γ, interferon gamma; rmTBI, repetitive mild traumatic brain injury; i.v., intravenous injection; Mn, manganese.

**Table 5:** Current and emerging neuromodulation approaches in neurorehabilitation with references to recent clinical studies of its use in brain injury rehabilitation.

Technique	Common Uses in Neurorehabilitation	Application in Brain Injury Rehabilitation
Non-Invasive Neuromodulation Techniques		
Transcranial Direct Current Stimulation (tDCS)	Applies low direct current to modulate cortical excitability. Used in stroke, cognitive rehab, and depression.	[180,181,182,183,184,185,186]
Transcranial Magnetic Stimulation (TMS/rTMS)	Uses magnetic fields to induce electric currents in the brain. Can excite or inhibit specific areas. Repetitive TMS (rTMS) is common in stroke and depression rehab.	[187,188,189,190,191]
Transcranial Alternating Current Stimulation (tACS)	Similar to tDCS but uses an alternating current. Targets neural oscillations (brain wave frequencies).	[192]
Transcranial Random Noise Stimulation (tRNS)	Applies random frequencies of current. Thought to increase cortical excitability and plasticity. Used for sensory-motor rehab, cognition.	[193]
Invasive or Semi-Invasive Neuromodulation		
Deep Brain Stimulation (DBS)	Surgically implanted electrodes. Often used in Parkinson’s, dystonia, and other movement disorders.	[194]
Epidural Cortical Stimulation	Electrodes placed over the dura (outer brain covering). Investigated in stroke recovery and epilepsy.	[195]
Vagus Nerve Stimulation (VNS)	Stimulates the vagus nerve (implanted or non-invasive versions exist). Used in epilepsy and increasingly in post-stroke motor recovery.	[196]
Emerging or Combined Techniques		
Closed-loop Neuromodulation	Real-time feedback systems that adjust stimulation based on brain activity (could be considered a bridge between neuromodulation and BMIs).	[197]
Paired Associative Stimulation (PAS)	Combines peripheral nerve stimulation with brain stimulation to boost synaptic plasticity. Used for motor learning and post-stroke.	[198]

**Table 6:** Proposed innovative approaches in brain trauma treatment that integrate molecular, cellular and technology-based strategies.

Molecular Strategies	Technology-Based Strategies	Mechanisms/Applications	Key Advancements
Gene Therapy	Nanoparticle Delivery	Delivering genes to modulate neuroprotection/regeneration	Nanoparticles cross the blood–brain barrier, enabling targeted gene delivery with reduced immune response
Stem Cell Therapy	Biomaterial Scaffolds	Enhancing stem cell survival and integration via 3D matrices	Hydrogels and nanocarriers improve stem cell engraftment, reducing inflammation and promoting repair
Exosome-based Therapy	Nanocarriers	Transporting exosomes or drugs to mitigate oxidative stress	Carbo-genic nanozymes and engineered exosomes show efficacy in modulating neuroinflammation
Precision Medicine	Brain–Computer Interfaces (BCI)	Using biomarkers (e.g., genomics) to tailor treatments	BCI enables neurofeedback for personalized cognitive rehabilitation, aligning with biomarker-guided plans

**Table 7:** Innovative brain trauma treatment approaches and their level of readiness for clinical implementation.

Treatment Approach	Readiness Level	Key Findings and Limitations
Nanomaterials for Targeted Delivery/Neuro-regeneration	Preclinical	Nanoparticles demonstrate efficacy in animal models for targeted drug delivery and neuroprotection but remain still in preliminary research phases for TBI. Clinical translation is limited due to scalability issues challenges in manufacturing (low reproducibility, high cost) and safety concerns (potential off-target accumulation in liver and lungs) and long-term toxicity concerns [206,209].
Cell-Based Therapies (Stem/Progenitor Cells)	Clinical (Phase I/II Trials)	Autologous bone marrow-derived MSCs show safety and efficacy in early-stage clinical trials, with improvements in consciousness and motor function observed in subacute TBI patients. Limitations include donor variability, risk of tumorigenicity, poor survival/engraftment, and high costs of autologous cell processing [207,210,211,212,213].
Precision Medicine	Preclinical/Early Clinical	Genetic risk factors (e.g., APOE, BDNF, Tau polymorphisms) are under investigation for patient stratification. No targeted therapies have reached late-stage clinical trials [7]. Limiting factors are that there are no TBI-specific biomarkers for patient stratification, the polygenic nature of TBI, and the fact that there are no approved therapies targeting genetic risk factors (e.g., APOE) [1].
Brain–Machine Interfaces (BMI) and Virtual Rehabilitation	Clinical (Pilot Trials)	Implantable BMI systems are in active clinical trials for restoring communication in patients with motor impairments (e.g., ALS, stroke). Virtual rehabilitation lacks TBI-specific clinical data but is emerging as an adjunct. Implementation issues are related different factors: signal drift/noise in chronic BMI use, limited TBI-specific validation for virtual rehab protocols, and high costs of BMI hardware/software [208].

Abbreviations: TBI, traumatic brain injury; MSCs, mesenchymal stem cells; APOE, apolipoprotein E; brain-derived neurotrophic factor; ALS, amyotrophic lateral sclerosis.

## Abbreviations

The following abbreviations are used in this manuscript:

AI	Artificial intelligence
APP	Amyloid precursor protein
APOE	Apolipoprotein E
AR	Augmented reality
BBB	Blood–brain barrier
BDNF	Brain-derived neurotrophic factor
BMI	Brain–machine interfaces
CT	Computed tomography
CTE	Chronic traumatic encephalopathy
DTI	Diffusion tensor imaging
FDA	U.S. Food and Drug Administration
GFAP	Glial fibrillary acidic protein
ICP	Intracranial pressure
iPSCs	Induced pluripotent stem cells
mTBI	Mild TBI
ML	Machine learning
MRI	Magnetic resonance imaging
MSCs	Mesenchymal stem cells
NPs	Nanoparticles
NfL	Neurofilament light chain
NSCs	Neural stem cells
NS/PCs	Neural stem/progenitor cells
QDs	Quantum dots
siRNA	Small interfering RNA
TBI	Traumatic brain injury
VR	Virtual reality
VRIs	Virtual rehabilitation interventions
UCH-L1	Ubiquitin carboxyl-terminal hydrolase L1
